# MAFb protein confers intrinsic resistance to proteasome inhibitors in multiple myeloma

**DOI:** 10.1186/s12885-018-4602-4

**Published:** 2018-07-06

**Authors:** Ya-Wei Qiang, Shiqiao Ye, Yuhua Huang, Yu Chen, Frits Van Rhee, Joshua Epstein, Brian A. Walker, Gareth J. Morgan, Faith E. Davies

**Affiliations:** Myeloma Institute, University of Arkansas for Medical Sciences, Winthrop P. Rockefeller Cancer Institute, 4301 West Markham St., Slot 776, Rm 914, Little Rock, AR 72205 USA

**Keywords:** MAF, Proteasome inhibitors, GSK3β, Caspases, Apoptosis, Myeloma, Drug resistance

## Abstract

**Background:**

Multiple myeloma (MM) patients with t(14;20) have a poor prognosis and their outcome has not improved following the introduction of bortezomib (Bzb). The mechanism underlying the resistance to proteasome inhibitors (PIs) for this subset of patients is unknown.

**Methods:**

IC50 of Bzb and carfilzomib (CFZ) in human myeloma cell lines (HMCLs) were established by MTT assay. Gene Expression profile (GEP) analysis was used to determine gene expression in primary myeloma cells. Immunoblotting analysis was performed for MAFb and caspase family proteins. Immunofluorescence staining was used to detect the location of MAFb protein in MM cells. Lentiviral infections were used to knock-down *MAFb* expression in two lines. Apoptosis detection by flow cytometry and western blot analysis was performed to determine the molecular mechanism MAFb confers resistance to proteasome inhibitors.

**Results:**

We found high levels of MAFb protein in cell lines with t(14;20), in one line with t(6;20), in one with Igλ insertion into *MAFb* locus, and in primary plasma cells from MM patients with t(14;20). High MAFb protein levels correlated with higher IC50s of PIs in MM cells. Inhibition of GSK3β activity or treatment with Bzb or CFZ prevented MAFb protein degradation without affecting the corresponding mRNA level indicating a role for GSK3 and proteasome inhibitors in regulation of MAFb stability. Silencing *MAFb* restored sensitivity to Bzb and CFZ, and enhanced PIs-induced apoptosis and activation of caspase-3, − 8, − 9, PARP and lamin A/C suggesting that high expression of MAFb protein leads to insensitivity to proteasome inhibitors.

**Conclusion:**

These results highlight the role of post-translational modification of MAFb in maintaining its protein level, and identify a mechanism by which proteasome inhibitors induced stabilization of MAFb confers resistance to proteasome inhibitors, and provide a rationale for the development of targeted therapeutic strategies for this subset of patients.

**Electronic supplementary material:**

The online version of this article (10.1186/s12885-018-4602-4) contains supplementary material, which is available to authorized users.

## Background

Multiple myeloma (MM) is a malignancy of terminally differentiated clonal plasma cells displaying significant molecular heterogeneity. The MF molecular subgroup defined by Gene Expression Profiling (GEP) at the University of Arkansas for Medical Sciences (UAMS) includes cases with overexpression of *MAFb* resulting from t(14;20) and *C-MAF* from t(14;16) [1]. Translocation t(14;20) occurs in approximately 1 to 2% of MM [2–4], and is associated with poor prognosis [3, 4] with the development of extramedullary disease [5] and primary plasma cell leukemia [6].

*MAFb* overexpression occurs as a result of t(14;20), in which genes at the 20q11 breakpoints are juxtaposed to IGH gene on 14q32 [4, 7, 8]. Additionally, high expression of the *MAFb* gene is also associated with translocation involving t(6;20) [7] or t(20;22) [9], or insertions of the 3’enhancers of Igλ or Igκ into the *MAFb* gene on 20q11 [10, 11]. Our previous work showed that t(4;14) and MF subgroup [including cases with a t(14:16) and t(14;20)] have an inferior survival [12, 13], and that the addition of Bortezomib (Bzb) to high dose melphalan based regimens provided a survival advantage to patients with t(4:14), while not benefitting patients in the MF subgroup [14, 15]. Previously, we have demonstrated that regulation of C-MAF stability by GSK3 is responsible for resistance to proteasome inhibitors (PIs) in t(14;16) MM [16]. It is currently unclear if a similar molecular mechanism is the cause for proteasome inhibitor resistance in patients with high MAFb expression.

The *MAFb* gene, one member of MAF family, is a basic leucine zipper transcription factor [17]. The MAFb protein may function as a transactivator or transrepressor depending on the target sequence of *MAF*-responsive elements and interacting proteins, such as c-Fos and ETS-1 [18, 19]. Amongst the MAF family, *MAFa* and *C-MAF* display the strongest oncogenic activity, whereas *MAFb* is less effective in transforming cells [20, 21], although *MAFb* can induce transformation of embryonic fibroblasts, and transgenic mice with *MAFb* expressed in hematopoietic stem cells develop plasma cell neoplasia [22]. In myeloma, overexpression of *MAFb* in U266 cells also enhances proliferation [23]. A number of target genes regulated by *MAFb* are shared with *C-MAF* target genes, including *Notch, CCND2, CCR1* and *ITGB7* [23, 24]. Up-regulation of cyclin D2 enhances cell cycle progression in t(14;16) cases [24, 25] and a high proliferative index [13, 26], whereas upregulation of ITGB7 and CCR1 stimulates MM growth via enhancing MM cell adhesion to stromal cells and cytokine secretion [27, 28].

Here, we demonstrate that high MAFb protein expression is associated with resistance to proteasome inhibitors, and demonstrate that similar to C-MAF mediated proteasome inhibitor resistance, protein stabilization underlies MAFb resistance to proteasome inhibitors. The results provide a rationale for the development of targeted therapeutic strategies directed at the treatment of this poor performing molecular subgroup.

## Methods

### Cell lines and primary CD138+ plasma cells

Human multiple myeloma cell lines (HMCLs) MM1S was purchased from ATCC (Manassas, VA, Catalogue number: CRL-2974). Other HMCLs were kind provided by Dr. Stuart Rudikoff, NCI, NIH and Dr. Michael Kuehl, NCI, NIH and described in detailed previously [16, 29, 30]. Cells were cultured in growth medium as described previously [29]. CD138-expressing patient MM cells were isolated as previously described [16].

### Gene expression analysis

*MAFb* gene expression was determined by GEP analysis of CD138-expressing MM cells from patients as described previously [1]. MAFb gene expression data set from primary CD138 expressing MM cell presented in this paper have been deposited in the NIH Gene Expression Omnibus (GEO; National Center for Biotechnology Information [NCBI], http://www.ncbi.nlm.nih.gov/geo/) under accession number GSE2658 as described previously [1].

### Silencing *MAFb* expression by short hairpin RNA

Knockdown of *MAFb* gene was performed in HMCLs expressing high levels of *MAFb* using a lentiviral expressing system [16]. Briefly, two sequences (5′ -CCGGGCCTTGTCTTATGGTCAAATTCTCGAGAATTTGACCATAAGACAAGGCTTTTT-3′ and 5′- CCGGGCCCAGTCTTGCAGGTATAAACTCGAGTTTATACCTGCAAGACTGGGCTTTTT-3′) specific to *MAFb* gene were engineered in lentiviral expressing system. A control oligonucleotide sequence not matching any sequence in the human genome (5′- CCGGTACAACAGCCACAACGTCTATCTCGAGATAGACGTTGTGGCTGTTGTATTTTT-3′) was used as a control shRNA sequence (designated as shCon). Total RNA, isolated after 24, 48, or 72 h, was subjected to reverse transcription (RT)-PCR and qPCR to determine of the degree of target gene silencing. Whole cell lysates were subjected to immunoblotting for MAFb protein measurement using a specific antibody.

### MTT assay for cell proliferation

The proliferation of MM cells was determined by colorimetric 3-(4, 5-dimethylthiazol-2-yl)-2, 5-diphenyltetrazolium bromide (MTT) assay [31]. Briefly, MM cells (4X10^4^/well) in 0.1 ml of IMDM medium with 10% FCS were cultured in flat-bottom, 96- well microliter wells. At the indicated time points, 10 ul of 5 mg/mL MTT was added to each well and incubated for 4 h at 37 °C, followed by adding 0.1 ml of 10% sodium dodecyl sulfate and incubation at 37 °C. Optical density was read on a Spectra Max340 Microplate Spectrophotometer (Molecular Devices) at 570 nm. Proteasome inhibitors including Bzb and Carfilzomib (CFZ) were used as described previously [32, 33].

### Detection of apoptotic cells

Cells were cultured in growth media alone for 24 h and treated with or without serial concentrations of proteasome inhibitors for indicated time points. The apoptotic cells were stained using Annexin V-FITC Apoptosis Detection Kit (BD Biosciences) as described previously [16]. Apoptotic cell numbers were detected by flow cytometry according to the manufacturer**’**s instructions and analyzed using BD FACSVerse™ System, (Becton Dickinson, Mountain View, CA) and BD FACSuite software. Assay for DNA content and cell cycle analysis was performed by FITC BrdU Flow Kit (BD Pharmingen™) staining following the manufacturer’s instructions and were analysized by FCS Express 5 Flow Cytometry Professional software (De Novo Software).

### Co-culture with bone marrow stromal cells

Co-culture of MM cells with human bone marrow stromal cells (HBMSCs) was performed to determine the effect of *MAFb* on MM apoptosis in the presence of the BM microenvironment as previously described [34]. MM cells were cultured on a bone marrow stromal layer in 6-well plates in the presence of serial concentrations of proteasome inhibitors (Bzb and CFZ) for 16 h. The MM cells were harvested and subjected to Annexin staining and apoptotic cells detected by flow cytometry analysis.

### Immunoblotting

Proteins were isolated from cells treated with or without Bzb, CFZ, MG132, cycloheximide (CHX), SB216763 [34], and LiCl for indicated time points prior to harvesting. The concentration of protein in each sample was determined by BCA protein Assay. Equal concentrations of total protein (50 μl per lane) were separated by SDS-PAGE followed by electrophoretic transfer to Immobilon polyvinylidene difluoride membranes. The proteins in the membranes were detected by specific antibodies including Anti-MAFb, caspase-3, − 6, − 7, − 8, and − 9, PARP, lamin A/C antibodies [31]. The membranes were stripped and reblotting with anti-actin antibody for indicating protein loading.

### Cycloheximide chase assay

For the study of MAFb half-life, cells were cultured in growth media in the presence of cycloheximide (CHX, 5 μg/ml, Sigma-Aldrich) for 0, 60, 120, 240 min and lysed at various time points. Cell lysate was subject to immunoblotting analysis. Protein loading was normalized by using anti-β-actin antibody. The autoradiographs were scanned and densitometry performed for quantification [33].

### Immunofluorescence staining

Immunofluorescence staining was used to determine MAFb protein expression in cell nuclei using a MAFb antibody and DAPI as described previously [32]. Cells were incubated with an anti-MAFb antibody after being fixed with 3.7% formaldehyde in PBS. The slides were stained with Alexa Fluor 488-labeled secondary anti-mouse IgG antibody or Alexa 594-labeled secondary anti-rabbit IgG antibody for visualization. All images were captured under an AxioImager fluorescent microscope (Zeiss, Jena, Germany) and digitalized with Zeiss AxioCam MRc5 and Zeiss Axiovision software.

### Real-time quantitative PCR

For quantitation of mRNA expression, total RNA was extracted from MM cell lines and cDNA was generated from 1μg RNA as described previously [33]. qPCR was performed using a QuantStudio™ 6 and 7 Flex Real-Time PCR Systems. The primers /probe sets including *MAFb* (Hs00271378_s1), *ITGB7* (Hs01565750-m1) and *CCR1* (Hs00174288-m1) were purchased from Life Technologies. The indicated genes were amplified using methods described in Additional file 1 and gene expression calculated using 2ΔCT analysis. [35, 36].

### Statistical analysis

The statistical significance of differences between experimental groups was analyzed by Student t test using the Microsoft Excel software. A significant *P* value was less than 0.05 by 2-tailed test.

## Results

### Expression of MAFb in primary myeloma and HMCLs

**Fig. 1:**
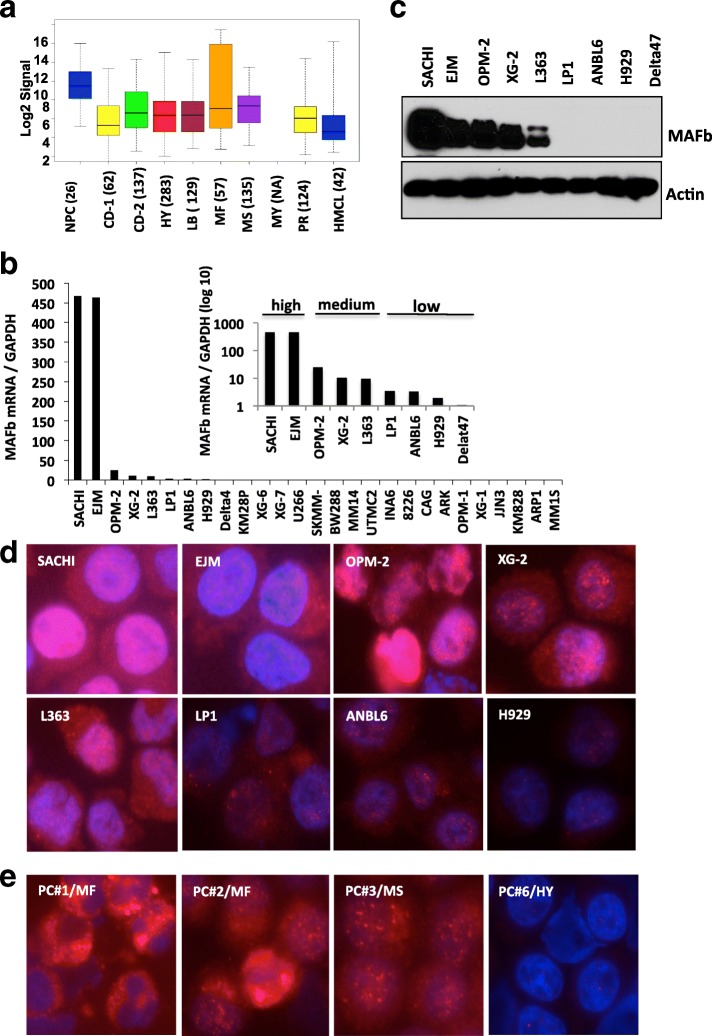
Expression of *MAFb* gene and protein in primary plasma cells and HMCLs. Affymetrix expression of *MAFb* mRNA was high in the GEP MF subgroup (**a**). Gene expression of 803 newly diagnosed MM patients was measured by U133 plus2.0 Affymetrix oligonucleotide microarray probe set 218559_s_at. The box plots indicate the middle of GEP signaling and dot lines with bars in black indicate quartiles. The cells were cultured in the growth mediate for 48 h and harvested for isolated RNA and *MAFb* mRNA in 27 HMCLs by qRT-PCR analysis as described in Material and methods (**b**). The cells were cultured in the growth mediate for 48 h and MAFb protein was measured in HMCLs by immunoblotting analysis described in Materials and Methods (**c**). MAFb protein in nuclei and cytoplasm of HMCLs (**d**) and MM PC from 4 patients (**e**) was determined with immunofluorescence staining with DAPI counterstaining. Images were taken with a fluorescence microscope with digital camera as described in supplemental data

We previously reported that patients in the MF subgroup including those with a t(14;20) have a poor survival and have not benefited from the addition of Bzb to their therapy [14, 15, 37]. We hypothesized that overexpression of MAFb protein confers intrinsic resistance of MM to proteasome inhibitors. To validate our hypothesis, we first assessed *MAFb* transcription in primary MM cells by examining *MAFb* mRNA from a panel of 803 patients belonging to several molecular subgroups using Affymetrix oligonucleotide microarrays. *MAFb* mRNA was highly expressed in 57 samples from patients in the MF subgroup, Fig. 1a. We next detected *MAFb* expression by q-RT-PCR in a panel of 32 HMCLs, Fig. 1b. The highest level of *MAFb* mRNA was seen in SACHI and EJM, both harboring t(14;20); intermediate levels of *MAFb* were seen in three cell lines; OPM-2 with t(4;14), XG2 with Eμ enhancer of immunoglobulin light chain (IGL) inserted near *MAFb* in 20q11 [10] and L363 with t(6;20) [7]. Low *MAFb* mRNA levels were seen in four lines; LP1, ANBL6, H929 and Delta47, while no MAFb mRNA was detected in the remaining cell lines.

Consistent with the mRNA levels in t(14;20), immunoblotting analysis demonstrated abundance of MAFb protein in SACHI and EJM, OPM-2 and XG-2 cells, with a low level in L363 cells, Fig. 1c. In contrast, MAFb protein was not detected in *MAFb* mRNA negative HMCLs. Comparable high levels of *MAFb* mRNA and protein were seen in SACHI, and EJM cells. Surprisingly, despite lower levels of *MAFb* mRNA, a relative high level of MAFb protein was seen in OPM-2 cells which harbors a t(4;14).

As mRNA were detected in some cell lines with no detectable protein by immunoblotting, immunofluorescent analysis was used to validate these findings. Weak expression of MAFb proteins was detected in LP1, ANBL6, and H929 cells, with high MAFb expression in SACHI, EJM, OPM-2, XG-2 and L363, Fig. 1d. These results suggest that in MM, immunofluorescent detection of MAFb protein is more sensitive than immunoblotting analysis. We therefore extended our analysis to CD138 selected MM cells using this sensitive method. We measured MAFb protein in CD138+ cells from 8 patients; two with t(14;20), two with t(4;14) and 4 belonging to other subgroups based on FISH analysis. The MAFb protein was highest in t(14;20), with intermediate levels in the two with t(4:14), and negative in the rest, Fig. 1e (data not shown). Taken together, these results indicate that MAFb protein abundance is likely correlated with the mRNA in t(14;20).

### High MAFb protein associated with resistance to proteasome inhibitors

**Fig. 2:**
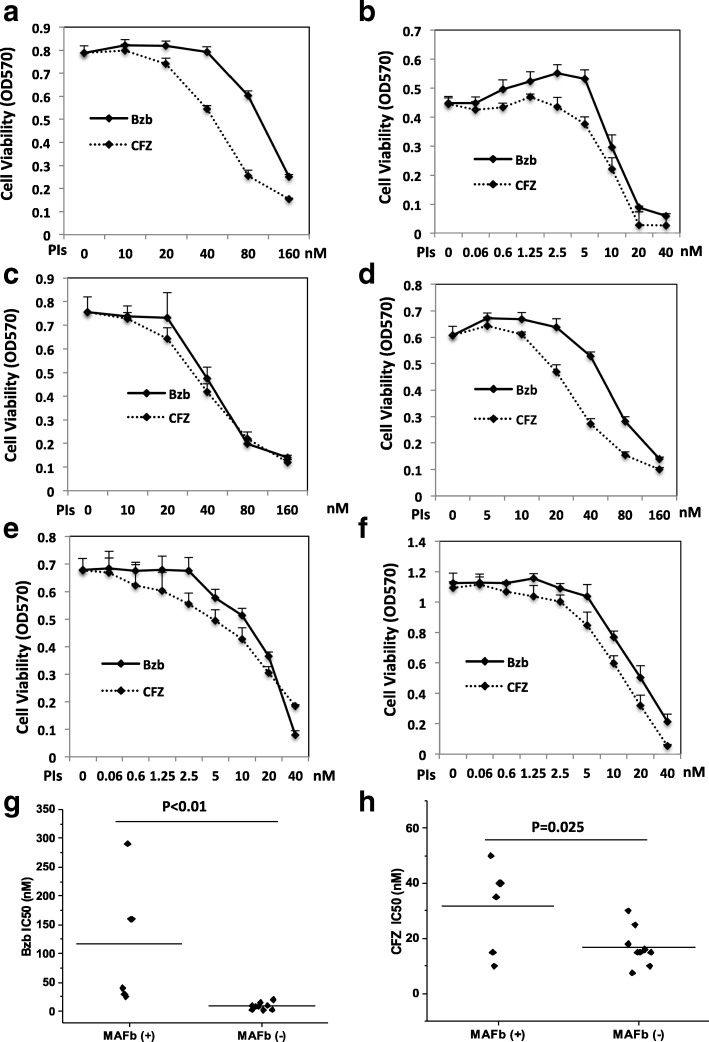
High MAFb expression is associated with resistance to PIs. SACHI (**a**), H929 (**b**), EJM (**c**), XG2 (**d**), L363 (**e**), and OPM-2 (**f**) cells were seeded at 2 × 10^4 per well in 96-well plates in the presence of indicated concentrations of Bzb or CFZ for 48 h. Cell survival was measured by MTT Assay. Results are presented as mean ± SE (*n* = 4). Data are representative of 3 separate experiments. HMCLs were treated with serial concentrations of Bzb (G) and CFZ (H) for 48 h and cell viability was measured by MTT assay. The IC50 of Bzb (G) and CFZ (H) were classified based on MAFb protein (+) or (−)

To investigate MAFb effects on the sensitivity of MM cells to proteasome inhibitors, we established the half-maximum inhibitory concentration (IC_50_) of Bzb and CFZ for five MAFb protein positive HMCLs and a number of t(14;20) negative HMCLs including H929 by MTT assay for 48 h. The IC_50_ of CFZ and Bzb for SACHI was 50- and 120-nM and, respectively, Fig. 2a. In contrast, treatment of H929 cells with 10-nM of Bzb or 6-nM of CFZ reached 50% of inhibition of cell proliferation, Fig. 1b. The IC_50_ of Bzb and CFZ for SACHI cells were 12- and 8.2-fold higher than for H929 cells, respectively. These results indicate that SACHI cells are more resistant to PIs than H929. Similarly, a relatively high IC_50_ for Bzb and CFZ was seen in EJM, Fig. 2c and XG2, Fig. 2d; respectively. The IC_50_ values of Bzb and CFZ for L363 cells were at a midlevel, Fig. 2e. The CI_50_ for Bzb and CFZ was 12- and 10-nM respectively, Fig. 2f, in OPM-2 cells despite similar high level of MAFb protein to SACHI and EJM. The mean IC_50_ of Bzb (Fig. 2g) and CFZ (Fig. 2h) were significantly lower than those in cells, which lacked MAFb expression. The IC50s of Bzb and CFZ in each tested human MM cell lines are summarized at Additional file 1: Table S1. Taken together, these results suggest that myeloma cells with high MAFb protein due to translocation to 20q11 or Igλ, or insertion on 20q11 are associated with resistance to proteasome inhibitors.

### MAFb protein is a fast-degraded protein

**Fig. 3:**
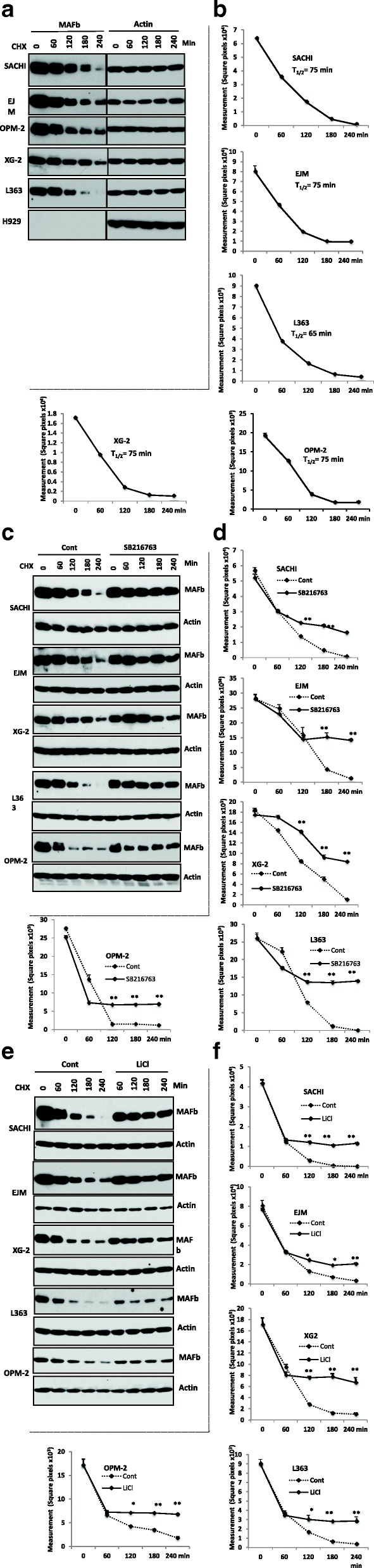
Inhibition of GSK3 activity by SB216763 stabilized MAFb protein. HMCLs were treated with 5 μg/ml of CHX for serial indicated time points to inhibit de novo protein synthesis. The MAFb protein was determined by immunoblotting analysis using anti-MAFb. The membranes were striped and reblotted with Anti-β-actin (**a**). The half-life of MAFb protein was determined by autoradiographs analysis using Adobe Photoshop software and NIH image software (**b**-**f**). HMCLs were treated with or without a specific GSK3 inhibitor, SB216763, at a concentration of 5 μg/ml for indicated times. MAFb protein was determined by immunoblotting analysis using anti-MAFb antibody. The membranes were striped and reblotted with Anti-β-actin to indicate protein loading (**a**). The protein decay curve is as described in Fig. 3b (b-f)

To determine if protein degradation regulates the level of MAFb protein, we used cycloheximide (CHX) to inhibit new protein synthesis from mRNA and determined the protein half-life [38]. As shown in Fig. 3a, MAFb protein decreases in the presence of CHX in 5/5 HMCLs by 60 min, while the actin protein level remains stable for each cell line. The decay curves show that the time for half the amount of MAFb protein to be degraded (T1/2) for 4/5 cell lines was 75 min, and 65 min for L363 cells, Fig. 3b. The half-life of MAFb protein was shorter than beta-actin protein. These results suggest that stability of MAFb protein in MM cells is regulated by post-translational modification. The MAFb protein appeared to become phosphorylated as indicated by a gel mobility shift (a slow migrating band shifted from the main band) at OPM2, XG2 and L363, Fig. 1c and at 180-min for SACHI Fig. 3a.

### Inhibition of GSK3 activity stabilizes MAFb protein.

Having demonstrated that protein degradation is responsible for MAFb protein levels in MM cells, we next sought to identify factors associated with regulating the stability of MAFb protein. Phosphorylation of MAF-A protein by GSK3 governs the degradation of the protein in other tissue as well as c-MAF in MM cells [16, 39]. To assess how the inhibition of GSK3 activity affects abundance of MAFb protein, we first used SB216763 to abrogate GSK3 activity. Treatment of HMCL with SB216763 led to blockage of degradation of MAFb protein, Fig. 3g. Significant stabilization of MAFb protein by SB216763 was observed by 120 min in SACHI, XG-2, L363, and OPM-2 cells, and by 180 min in EJM, Fig. 3 (h to l), indicating that activity of GSK3β is required for degradation of MAFB protein. To confirm that GSK3 activity affects MAFb stability, we treated the cell lines with LiCl, another GSK3beta inhibitor. Exposure to LiCl led to a stabilization of MAFb protein in all 5 MM cell lines within 120 min, (Additional file 1: Figure S1). These results confirm that GSK3 activity is required for degradation of MAFb protein in MM. Since MAPK regulates stability of MAF protein in other cell type [40], we determined if MAPK is involved in regulating stability of MAFb protein. To do so, we utilized U0126, which inhibits subsequent phosphorylation and activation of ERK by MEK [31]. Treatment of SACHI, EJM and XG-2 cells with U0126 did not affect MAFb protein levels (data not shown). We next looked at the effect of p38 and Jun N-terminal protein kinases on stabilization of MAFb protein by treatment of these cells with SB20350, a well-known, p38 inhibitor and SP6000125, a Jun N-terminal protein kinases, alone or in combination. Neither SB20350 alone, nor SP6000125 alone, nor combination of these two inhibitors together affect the level of MAFb protein, respectively (data not shown). Taken together, these results suggest that GSK3 activity regulates degradation of MAFb protein in MM cells independently of the MAPK pathway and p38 kinases.

### Proteasome inhibitors stabilizes MAFb protein

**Fig. 4:**
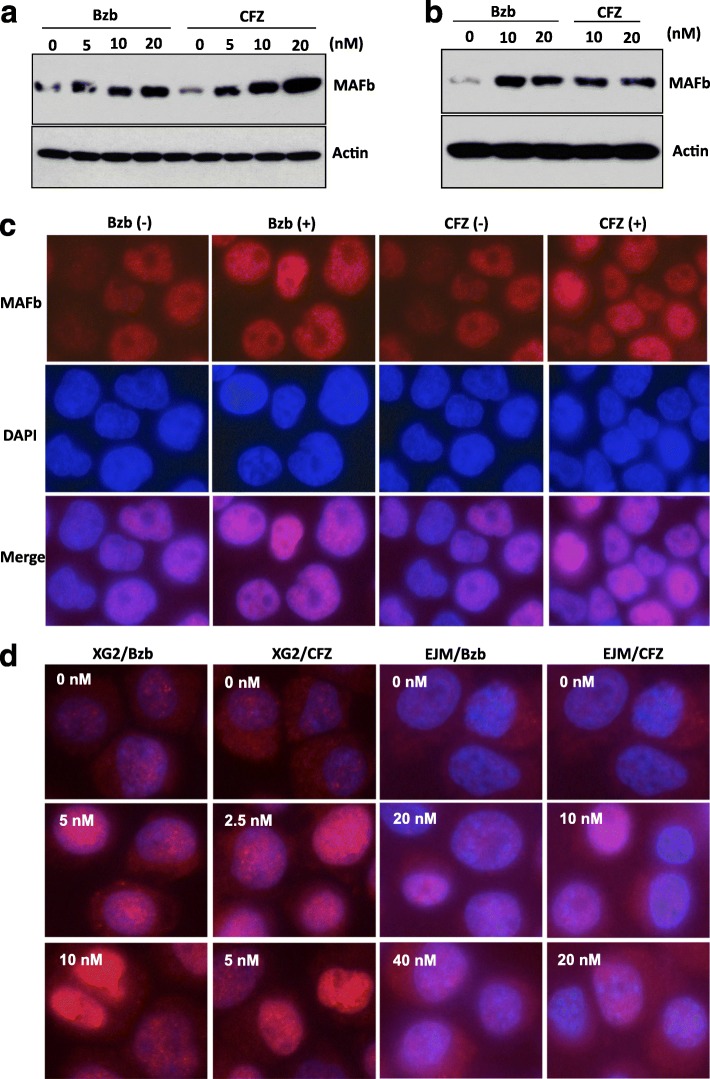
Proteasome Inhibitors stabilize MAFb protein. The SACHI (**a**) and XG2 (**b**) cells were treated with indicated concentrations of Bzb or CFZ at the presence of 10 μg/ml of CHX for 6 h. MAFb protein lysate was analyzed as described in Fig. 2. SACHI (**c**) were treated with 20-nM of Bzb or CFZ for 12 h; XG-2, and EJM cells (**d**) were treated with indicated serial concentrations of Bzb and CFZ for 12 h, MAFb protein in nuclei and cytoplasm of the cells was determined by analysis of immunofluorescence staining with DAPI counterstaining to indicate the nucleus. Images were taken with a fluorescence microscope with digital camera as described in Methods

Having shown MAFb protein is regulated by GSK3 activity, we next sought to see if the proteasome inhibitors had an effect on the stability of MAFb protein. Treatment of SACHI cells with Bzb resulted in an increase in MAFb, Fig. 4a. An increase in MAFb was also observed following treatment with CFZ. A similar effect of Bzb and CFZ on MAFb protein was observed in XG-2, Fig. 4b, while the proteasome inhibitors had no effect on MAFb mRNA level by qPCR analysis (data not shown) suggesting that proteasome inhibitors prevent degradation of MAFb in a dose-dependent manner.

Since MAFb is a transcription factor [41], we determined the effect of proteasome inhibitors on localization of MAFb protein using immunofluorescent staining. Treatment of SACHI cells with 20-nM of Bzb and CFZ led to an increase of MAFb in the nucleus, Fig. 4c. Similar increases in MAFb in the nucleus and cytoplasm were seen in XG-2 and EJM in response to Bzb and CFZ, Fig 4d, while no increase MAFb protein was seen in OPM-2 cells (data not shown) although inhibition of GSK3 activity stabilized MAFb protein in this cell line. Taken together, these results suggest that blockade of proteasome activity by proteasome inhibitors prevents degradation of MAFb protein in the cells with t(14;20) or Igλ insertion, independent of mRNA regulation.

### Knockdown *MAFb* expression restores sensitivity to proteasome inhibitors

**Fig. 5:**
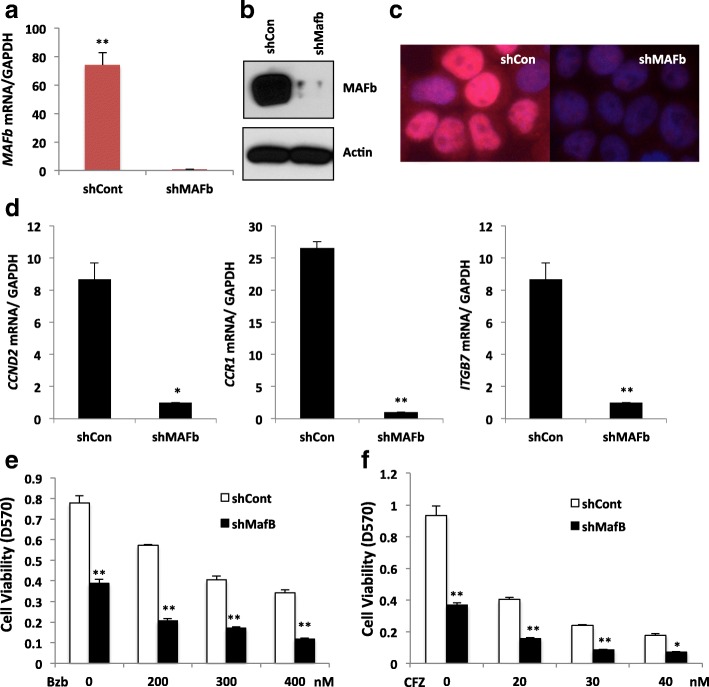
Knockdown of *MAFb* restores sensitivity of myeloma to PIs. SACHI cells were infected with lentiviral expression system containing shRNA specific to *MAFb* gene (sh*MAFb*) or shRNA containing scramble sequences (shCon) for 48 h. *MAFb* mRNA was measured by RT-qPCR analysis (**a**), MAFb protein in whole lysis buffer was analyzed by immunoblotting analysis (**b**) and in both cytoplasmic and in nucleus by immunofluorescent staining analysis (**c**) as described in Fig. 5d. *MAFb* target genes in sh*MAFb* and shCon cells were analyzed by qRT-PCR analysis (**d**). The cells were treated with indicated concentrations of Bzb (**e**) or CFZ (**f**) for 48 h and cell survival was measured by MTT assay. Results are presented as mean ± SE (*n* **=** 4). Data are representative of 3 separate experiments. *P < 0 .01 versus control. **P < 0 .001 versus control

To validate the ability of MAFb-mediated resistance to proteasome inhibitor, loss-of-function studies were undertaken using SACHI and EJM cells with robust MAFb expression. To reduce *MAFb* level, we used shRNA to reduce *MAFb* expression by 90% compared to cells infected with a scrambled shRNA (shCon), Fig. 5a. A decrease in MAFb protein level in sh*MAFb* cell was observed with an associated decrease in nuclear expression, Fig. 5b and c. Upon MAFb knockdown, a reduction in expression of *MAFb* target genes, including *CCR1, ITGB7* and *CCND2* was evident, indicating a functional *MAFb* knockdown, Fig. 5d. We further explored the effect of *MAFb* knockdown on proliferation of SACHI cells, and found that proliferation in *MAFb* knockdown cells markedly reduced, Fig 5e. Furthermore, we evaluated the effect of knockdown of MAFb protein on sensitivity to proteasome inhibitors. In the presence of 200-, 300- and 400-nM of Bzb, the proliferation of sh*MAFb* cells was significantly lower than those in shCon indicating knockdown of *MAFb* restores sensitivity to proteasome inhibitors. The effect of silencing *MAFb* on sensitivity to proteasome inhibitors was more obvious with CFZ, Fig. 5f. A similar increase in sensitivity to Bzb was seen in EJM upon knockdown of *MAFb* (data not shown). It should be noted that MM cell infected with lentivirus particle to expressing shCon or shMAFb lead to approximate 5% of cell undergoing apoptosis shCon and shMAF, compared with the cell without infected with lentivirus particle at the absence of treatment of proteasome inhibitors (data not shown). These results suggest that high expression of MAFb protein confers the intrinsic resistance of MM to proteasome inhibitors.

### Silencing MAFb enhances proteasome inhibitor-induced apoptosis

**Fig. 6:**
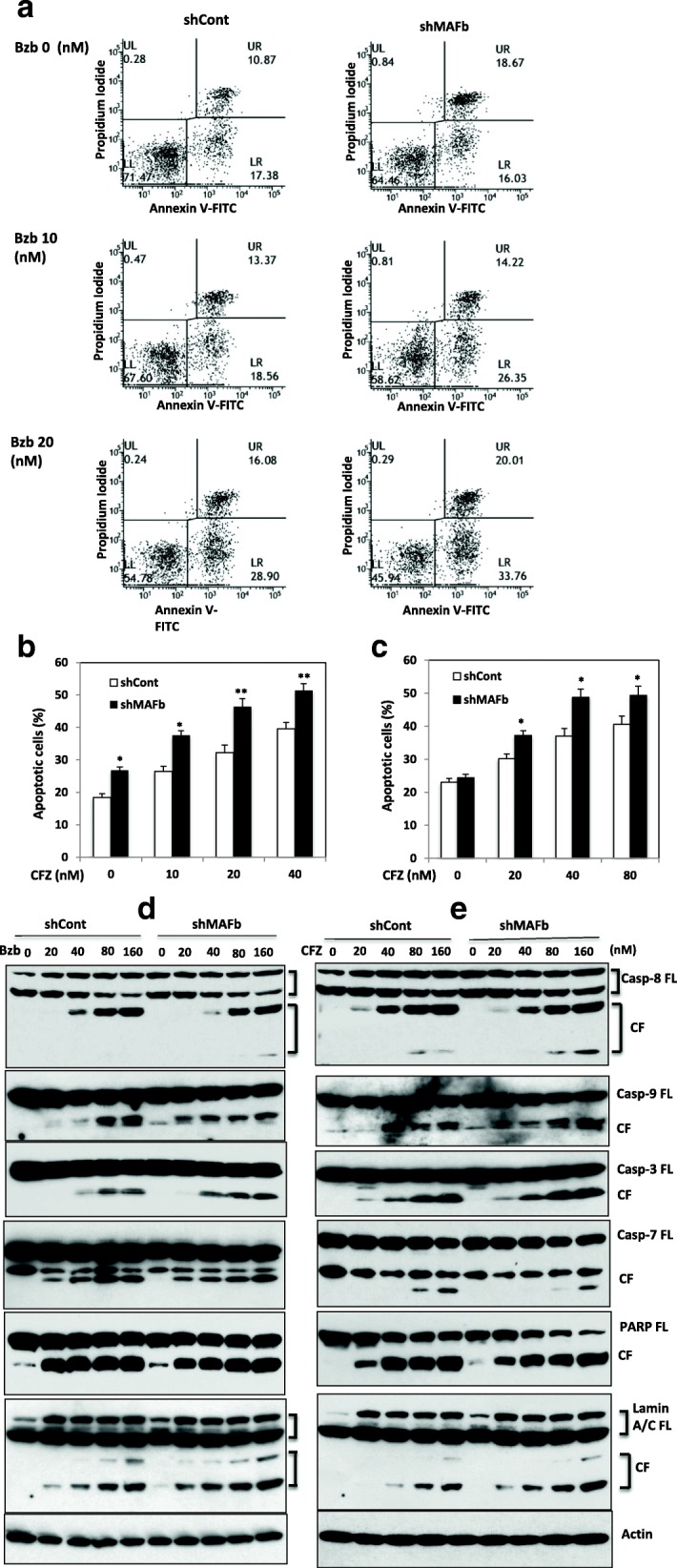
Silencing *MAFb* enhanced PIs-induced apoptosis and activation of caspases. SACHI/sh*MAFb* or SACHI/shCon cells were treated with serial concentrations of Bzb (**a**) or CFZ (**b** and **c**) with (**c**) or without HBMSC (**a** and **b**) for 14 h and apoptotic cell numbers determined by Annexin V staining and flow cytometry analysis as described in supplemental data. Results are presented as mean ± SE (*n* **=** 3). *P < 0 .01 versus control. **P < 0 .001 versus control. SACHI/sh*MAFb* and SACHI/shCon cells were treated with indicated concentrations of Bzb (A) or CFZ (**b**) for14 hours. Protein was resolved in SDS-PAGE. The full-length (FL) and cleavage fragments (CFs) of each indicated protein were determined by immunoblotting analysis using antibodies specifically recognizing of caspases-3, − 7, − 8 and − 9, PARP and lamin A/C

The effect of knockdown of MAFb on proteasome inhibitors-induced apoptosis in sh*MAFb/*SACHI and shCon/SACHI cells was determined using Annexin V staining and flow cytometry analysis. Increases in the number of apoptotic cells were seen in sh*MAFb* cells treated with Bzb, Fig. 6a and by CZF, Fig. 6b. These results indicate that silencing *MAFb* gene and protein enhances proteasome inhibitor-triggered apoptosis.

To see if the BM microenvironment affects the role of MAFb in proteasome inhibitor-mediated apoptosis, sh*MAFb* and shCon cells were co-cultured with primary bone morrow stromal cells from myeloma patients. Treatment of sh*MAFb* cells with CFZ led to increases in apoptotic cells at the presence of co-culture with bone marrow stromal cells, Fig. 6c, suggesting that interaction with bone marrow microenvironment does not protect MM cells from proteasome inhibitor-induced apoptosis.

### Silencing *MAFb* enhances proteasome inhibitor-induced activation of caspases

Since the caspase family plays an important role in apoptosis [42], we next determined whether specific caspases regulated proteasome inhibitor-induced apoptosis in MAFb knockdown cells. We used Western blot to analyze the inactive as well as the cleaved, active forms of caspases in the loss-of-function (sh*MAFb* and shCon) cells exposed to Bzb and CFZ. The protein levels of the cleavage fragment of caspases-8, an initiator caspases that functions as an active executioner of the caspase pathway [42], in sh*MAFb*/SACHI were higher than those in shCon/SACHI cells, Fig. 6d. An increase in of caspases-9, another initiator of caspases, was observed in sh*MAFb*/SACHI cells, without Bzb treatment indicating that silencing *MAFb* itself induces MM cell apoptosis. Importantly, an obvious increase in the cleavage fragment of caspase 9 was seen at 20 min and maintained for 160 min after to Bzb treatment in sh*MAFb* cells. These results indicate that Bzb-induced apoptosis is mediated via activation of caspases-8 and -9, and knockdown MAFb enhances Bzb-induced activation of caspases-8 and -9 in MM cells.

Because caspases-3, − 6 and − 7 proteins are substrates of active caspase-8 and -9 [42], we examined the cleavage products in both sh*MAFb* and shCon cells. Increases in cleavage fragment of caspase 3 in sh*MAFb* cells were seen in the cells following treatment with Bzb, Fig. 6e. In contrast, silencing MAFb did not affect activation of caspases-7 and -6 (middle panel and data not shown). These results indicate MAFb protein diminishes Bzb-induced activation of caspases-3 but not caspase-6 and -7.

We next determine whether MAFb affects Bzb-induced activation of PARP, which is one of the major substrates of activated caspase-3 [43]. The cleavage of PARP facilitates cellular disassembly and serves as a hallmark of cells undergoing apoptosis [44]. Increases in the cleavage fragment of PARP were observed in sh*MAFb*/SACHI cells. Moreover, the cleavage fragment of PARP was increased following treatment with Bzb. Thus, knockdown of *MAFb* promotes Bzb-induced activation of PARP.

Lamins are major factors in the structural organization and function of the nucleus and chromatin and cleavage of lamins results in nuclear deregulation and cell death [45]. We investigated the effect of MAFb protein on activation of lamins in response to Bzb. An increase in cleavage fragments of lamin A/C in sh*MAFb* cells was clearly seen with Bzb, Fig 6d. Similar increases in cleavage fragments of caspases-3, − 7, − 8, and − 9, and PARP and lamin A/C were observed in sh*MAFb* cells response to CFZ, Fig. 6e. Therefore, knockdown of *MAFb* was accompanied by increased activation of caspases − 8 and − 9, which in turn induced activation of caspase-3, and at the end resulting in the degradation of PARP and lamin A/C in response to the proteasome inhibitors.

## Discussion

In this study, we demonstrate that cell lines with high levels of MAFb protein due to t(14;20) or Igλ insertion of 20q11 are resistant to Bzb and CFZ, and the lack of sensitivity of these cell lines to Bzb or CFZ is similar to HMCLs harboring a t(14;16) which have high C-MAF levels [16]. In contrast, HMCLs with t(4;14) or t(11;14) were more sensitive to Bzb and CFZ. This observation is consistent with clinical studies in which patients in the MF subgroup including patients with t(14;20)/MAFb [1] did not benefit from the addition of the Bzb in contrast to t(4;14) cases which gained a major advantage [14, 15, 37].

An assessment of the mechanisms by which t(14:20) patients with high MAFb expression are resistant to PIs is critical to understanding the biology of this subgroup and may provide a basis for targeted therapeutic approaches. The mechanisms that control the *MAFb* transcription in MM have been investigated by several laboratories [7, 8, 10, 11, 46]. It is well recognized that deregulation of *MAFb* transcription in t(14;20) patients results from juxtaposing the *MAFb* gene with the strong enhancer of the IgH locus, leading to overexpression of *MAFb* mRNA [7, 8]. Insertion of Igλ into *MAFb* locus is also associated with high transcription of *MAFb* in t(14;20) negative myeloma [10]. Vatsveen et al. reported that the cell line OH-2 that does not have an IGH translocation but has a complex translocation involving the *IgK* locus juxtaposed with both *MYC* and *MAFb,* expresses *MAFb* mRNA at a level that is higher than the three HMCLs SACHI, EJM and SKMM-1 that have t(14;20) [11]. Consistent with the high levels of *MAFb* mRNA, we found high levels of MAFb protein in SACHI and EJM. These results are in keeping with a study that shows MAFb protein in the nucleus of plasma cells in BM biopsies of MM patients with t(14;20) by immunohistochemistry [47]. When screening 32 MM cells lines, we found a high MAFb protein level in OPM-2 which harbors a t(4;14). This cell line has no cytogenetic abnormality involving in 20q11 or *MAFb* gene, suggesting an inconsistency between the gene transcriptional level and protein level of MAFb in t(4;14) cells. The high MAFb protein without comparable MAFb transcriptional levels suggests that post-transcriptional/translational regulation can govern the abundance of MAFb protein. In support our observation, Herath et al. have reported that MAFB and c-MAF are phosphorylated by GSK3eta in human MM cells [48].

Many proteins including transcriptional factors are known to be substrates of GSK3 [49]. Our results demonstrate that inhibition of GSK3 activity led to the accumulation of MAFb protein. Furthermore, both Bzb and CFZ induce stabilization of MAFb protein in a dose-dependent manner in MM cells with t(14;20) or t(6;20) or Igλ insertion in 20q, associated with accumulation of MAFb in nucleus and cytoplasm. Thus, PIs play a key role of regulating the stability of MAFb protein in MM cells. It should be noted that proteasome inhibitor-induced stabilization of MAFb protein did not occur in the OPM-2 cells with a t(4;14) despite high endogenous MAFb protein levels, and that inhibition of GSK3 activity prevented degradation of MAFb protein in OPM-2 cells. Indeed, the IC50 of Bzb and CFZ for OPM-2 and other cell lines with a t(4;14) were much lower than those with t(14;20), suggesting a difference in the degree of GSK3 activity between the t(14;20) and OPM-2 cells. In agreement with this hypothesis, several studies have reported that MM cells which harbor a deletion of PTEN have constitutive activation of AKT and p70S6 kinase [50, 51], which in turn leads to constitutive phosphorylation and inactivation of GSK3 [52]. As a consequence, inactivity of GSK3 results in endogenous high levels of MAFb protein in OPM-2 cells which have a PTEN mutation [50].

Clinical studies on the prognostic value of t(14;20) translocation and high expression MAFb indicate occurrence of this t(14;20) or high expression MAFB is correlated with poor prognosis and resistance to proteasome inhibitors. Boersma-Vreugdenhil et al. [7] reported that the t(14;20) is associated with short survival time after diagnosis. Studies on MAFb gene expression by GEP analysis indicates that MF subgroup including high expression of MAFb together with C-MAF signatures belongs to high risk group [1] and associated with poor overall survival [12, 13] and patients in the MF subgroup associated with resistance to Bzb [14, 15].

## Conclusions

In summary, high levels of MAFb protein in MM cells with a translocation involving q20 or Igλ insertion are associated with resistance to proteasome inhibitors. MAFb protein confers intrinsic resistance of MM cells to proteasome inhibitors via the abrogation of proteasome inhibitor-induced apoptosis and activation of the caspase family. The elucidation of the mechanism underlying MAFb-mediated resistance to proteasome inhibitors in myeloma with t(14:20) or Igλ insertion is important in the understanding of MM biology and may help in providing novel therapeutic approaches for targeting this rare subgroup of patients.

## Additional file

Additional file 1: Supplementary data. **Figure S1.** described Exposure to LiCl, an inhibitor of GSB3beta led to a stabilization of MAFb protein in all 5 MM cell lines within 120 min. The half-maximum inhibitory concentration (IC_50_) of proteasome inhibitors, Bzb and CFZ in each tested human MM cell lines are summarized at **Table S1.** (DOCX 812 kb)

## Abbreviations

Bzb: Bortezomib
*CCND2*: *CyclinD2*
*CCR*: *C-C: Chemokine receptor type1*
CFZ: Carfilzomib
GEP: Gene Expression Profiling
HBMSCs: Human bone marrow stromal cells
HMCLs: Human myeloma cell lines
Ig: Immunoglobulin
*ITGB7*: *Integrin beta7*
MM: Multiple myeloma
MTT: 3-(4, 5-dimethylthiazol-2-yl)-2, 5-diphenyltetrazolium bromide
PIs: Proteasome inhibitors
