# Supplementary material for: MAFb protein confers intrinsic resistance to proteasome inhibitors in multiple myeloma
Source: BMC Cancer. 2018 Jul 6;18:724. doi: 10.1186/s12885-018-4602-4 (PMC6035431; doi:10.1186/s12885-018-4602-4)
Supplement: Supplementary file 1 — Supplementary data. Figure S1. described Exposure to LiCl, an inhibitor of GSB3beta led to a stabilization of MAFb protein in all 5 MM cell lines within 120 min. The half-maximum inhibitory concentration (IC50) of proteasome inhibitors, Bzb and CFZ in each tested human MM cell lines are summarized at Table S1. (DOCX 812 kb) [file 12885_2018_4602_MOESM1_ESM.docx]

**Supplementary Data**

**Figure S1. Inhibition of GSK3 activity by** LiCl **stabilized MAFb protein**. HMCLs were treated with or without a specific GSK3 inhibitor, LiCl, at a concentration of 10 mM for indicated times. MAFb protein was determined by immunoblotting analysis using anti-MAFb antibody. The membranes were striped and reblotted with Anti-β-actin to indicate protein loading (A). The half-life of MAFb protein was determined by autoradiographs analysis using Adobe Photoshop software and NIH image software.

**Table S1: IC50 of proteasome inhibitors**

| **HMCL** | **Bzb(nM)** | **CFZ(nM)** |
| --- | --- | --- |
| **SACHI** | **120** | **50** |
| **EJM** | **40** | **40** |
| **OPM-2** | **12** | **10** |
| **XG-2** | **50** | **40** |
| **L363** | **20** | **25** |
| **H929** | **10** | **6** |

HMCI: human myeloma cell line; Bzb: bortizomib; CFZ: carfilzomib
